# Long-term safety of OnabotulinumtoxinA treatment in chronic migraine patients: a five-year retrospective study

**DOI:** 10.3389/fneur.2024.1417831

**Published:** 2024-06-13

**Authors:** María Pilar Navarro-Pérez, Vicente González-Quintanilla, Albert Muñoz-Vendrell, Elisabet Madrigal, Alicia Alpuente, Germán Latorre, Francis Molina, María José Monzón, Vicente Medrano, David García-Azorín, Carmen González-Oria, Ana Gago-Veiga, Fernando Velasco, Isabel Beltrán, Noemí Morollón, Javier Viguera, Javier Casas-Limón, Jaime Rodríguez-Vico, Elisa Cuadrado, Pablo Irimia, Fernando Iglesias, Ángel Luis Guerrero-Peral, Robert Belvís, Patricia Pozo-Rosich, Julio Pascual, Sonia Santos-Lasaosa

**Affiliations:** ^1^Hospital Clínico Universitario Lozano Blesa, Zaragoza, Spain; ^2^Institute for Health Research Aragon and University of Zaragoza, Zaragoza, Spain; ^3^Hospital Universitario Marqués de Valdecilla, Universidad de Cantabria e IDIVAL, Santander, Spain; ^4^Hospital de Bellvitge, Barcelona, Spain; ^5^Hospital Universitario de Burgos, Burgos, Spain; ^6^Headache Clinic, Neurology Department, Vall d’Hebron Hospital, Barcelona, Spain; ^7^Headache and Neurological Pain Research Group, Vall d’Hebron Research Institute, Barcelona, Spain; ^8^Hospital Universitario de Fuenlabrada, Universidad Rey Juan Carlos, Madrid, Spain; ^9^Hospital Universitario Son Espases, Palma de Mallorca, Spain; ^10^Hospital Clínico Universitario Miguel Servet, Zaragoza, Spain; ^11^Hospital General Universitario de Elda, Alicante, Spain; ^12^Hospital Clínico Universitario de Valladolid, Valladolid, Spain; ^13^Hospital Clínico Universitario Virgen del Rocío, Sevilla, Spain; ^14^Hospital Universitario La Princesa, Madrid, Spain; ^15^Hospital Universitario de Cruces, Bilbao, Spain; ^16^Hospital General Universitario de Alicante, Alicante, Spain; ^17^Headache and Neuralgia Unit, Department of Neurology, Hospital de La Santa Creu I Sant Pau, Barcelona, Spain; ^18^Unidad Clínica de gestión de Neurociencias, Hospital Universitario Virgen Macarena, Sevilla, Spain; ^19^Hospital Universitario Fundación de Alcorcón, Madrid, Spain; ^20^Hospital Universitario Fundación Jiménez Díaz, Madrid, Spain; ^21^Hospital del Mar, Barcelona, Spain; ^22^Neurology Department, Clínica Universidad de Navarra, Pamplona, Spain

**Keywords:** chronic migraine, migraine, OnabotulinumtoxinA, prevention, prophylaxis, treatment

## Abstract

**Background:**

Real-world studies have shown the sustained therapeutic effect and favourable safety profile of OnabotulinumtoxinA (BoNTA) in the long term and up to 4 years of treatment in chronic migraine (CM). This study aims to assess the safety profile and efficacy of BoNTA in CM after 5 years of treatment in a real-life setting.

**Methods:**

We performed a retrospective chart review of patients with CM in relation to BoNTA treatment for more than 5 years in 19 Spanish headache clinics. We excluded patients who discontinued treatment due to lack of efficacy or poor tolerability.

**Results:**

489 patients were included [mean age 49, 82.8% women]. The mean age of onset of migraine was 21.8 years; patients had CM with a mean of 6.4 years (20.8% fulfilled the aura criteria). At baseline, patients reported a mean of 24.7 monthly headache days (MHDs) and 15.7 monthly migraine days (MMDs). In relation to effectiveness, the responder rate was 59.1% and the mean reduction in MMDs was 9.4 days (15.7 to 6.3 days; *p* < 0.001). The MHDs were also reduced by 14.9 days (24.7 to 9.8 days; *p* < 0.001). Regarding the side effects, 17.5% experienced neck pain, 17.3% headache, 8.5% eyelid ptosis, 7.5% temporal muscle atrophy and 3.2% trapezius muscle atrophy. Furthermore, after longer-term exposure exceeding 5 years, there were no serious adverse events (AE) or treatment discontinuation because of safety or tolerability issues.

**Conclusion:**

Treatment with BoNTA led to sustained reductions in migraine frequency, even after long-term exposure exceeding 5 years, with no evidence of new safety concerns.

## Background

Based on the 2021 Global Burden of Disease Study, migraine is considered one of the most common disabling neurological condition and the third leading cause of disabilities worldwide (1). Chronic migraine (CM) is defined as a headache that occurs 15 or more days per month for more than 3 months, which, on at least 8 days/month, has the features of migraine headache (2). It is estimated that CM affects 1–4% of the population and that 2.2–3.1%of patients with episodic migraine progress to CM every year (3).

BoNTA is one of the few approved treatments for the prophylaxis of CM in adults. The efficacy of BoNTA was established in large multicentre randomised clinical trials: Phase III Research Evaluating Migraine Prophylaxis Therapy (PREEMPT) 1 and 2 (4, 5) and in several subsequent studies (6–11).

Real-world studies have proven the sustained therapeutic effect and favourable safety profile of BoNTA in the long term and up to 4 years of treatment (12–20). This study aims to assess the safety profile of BoNTA in CM after 5 years of treatment in a real-life cohort of patients. As a secondary objective, we also aim to evaluate the efficacy and impact of this treatment on acute medication intake.

## Methods

This multicentre prospective study included patients who underwent 5 or more years of treatment with BoNTA that were followed in headache clinics in Spain.

### Patients

We included patients from 19 headache clinics who were visited consecutively for 4 months (from October 2020 to February 2021) and who had been treated with BoNTA for at least 5 years. After asking for their consent, we recorded their current migraine frequency status and performed a retrospective chart review of their baseline status.

The inclusion criteria were as follows: (1) adult (≥18 years old) at baseline; (2) CM diagnosis according to the International Classification of Headache Disorders (ICHD-3) criteria (2); and (3) history of inadequate response to at least two oral prophylactic migraine treatments (one of which had to be topiramate if no contraindications for its use or after tolerability failure, were noted based on our national guidelines) (21). The overuse of acute medication and continuation of a stable dose of concomitant oral prophylactic therapy were allowed.

The study was conducted in accordance with the STROBE guidelines for observational studies (22) and Declaration of Helsinki. Ethics approval was obtained from the Ethics Review Board of Zaragoza Health Area (GEC-ONA-2019-01) based in the Aragón Institute for Health Research (IIS Aragón), Zaragoza, Spain.

### Data collection

The patients’ data were collected, including demographics (age, sex), migraine characteristics (i.e., type of migraine, age at migraine onset, duration of CM, analgesic overuse and preventive treatments), and BoNTA treatment characteristics (i.e., number of cycles and injection interval).

Safety and tolerability were assessed by reviewing the frequency and nature of the AE, which were determined at the corresponding visits through patient self-report, general non-directed questioning and physical examination.

The efficacy data included the reduction in Monthly Headache Days (MHDs), Monthly Migraine Days (MMDs), acute migraine medication days per month and mean headache attack intensity (measured based on the reported score using a visual analogue scale). Patient treatment responsiveness in terms of relative reduction for the number of MHDs and MMDs with respect to the baseline values was evaluated. We classified the patients as follows: non-responder (<30% reduction), partial responder (≥30 and < 50% reduction), responder (≥50 and ≤ 75% reduction) and high responder (>75% reduction). Moreover, we proceeded in the same way with the improvement of mean pain intensity.

Data were collected from the patients’ medical records at the baseline visit and the trimester after completing 5 or more years of treatment.

### Data and statistical analysis

Statistical analysis was performed using the IBM SPSS Statistics 20.0 software for Mac (SPSS Inc., Chicago, IL, United States). The patients’ characteristics were reported as mean ± standard deviation (SD) or median and interquartile range (IQR) and percentages for the continuous and categorical variables, respectively. The Kolmogorov–Smirnov method was applied to confirm that the data were sampled from a Gaussian distribution. Moreover the differences between the effectiveness of treatment variables were compared using a two-paired t-test. Furthermore, statistical significance was set at a *p*-value of <0.05.

## Results

A total of 489 patients were included [mean age, 49 ± 13.7; 82.8% female]. Patients who discontinued treatment due to lack of efficacy or poor tolerability were excluded. The patients had a mean age of onset of migraine of 21.8 ± 5.6 (18–85) years and CM for an average of 6.4 ± 3 years; 20.8% of them fulfilled the aura criteria for migraine. At baseline, the patients reported a mean of 24.7 ± 5.8 MHDs and 15.7 ± 5.9 MMDs. The main pain intensity (*n* = 460) was 8.9 ± 1.01. Moreover, 66.4% were diagnosed with medication overuse headache. The patients had failed a mean of 4.2 ± 1.9 preventive treatments at baseline. The most frequently used drugs were topiramate (91.4%), amitriptyline (79.3%) and beta-blockers (70.4%). Table 1 shows the patients’ baseline demographic and treatment characteristics. All patients received at least 20 (25.8 ± 5.7; 20–37; median, 23; IQR, 20–27) cycles of BoNTA (62.8% quarterly, 28.3% every 4 months and 7.3% biannually) with a mean dose of 181.8 ± 33.3 U (100–400) (median, 195 U; IQR, 155–200).

**Table 1:** Demographic and clinical characteristics of chronic migraine patients.

Variables	Mean ± SD/percentage
Age (years)	49 ± 13.7
Gender (women)	82.8%
Aura	20.8%
MOH	66.4%
Number of non-steroidal anti-inflammatory drugs (*n* = 418)	18.7 ± 13.5
Number of triptans per month (*n* = 449)	9.0 ± 7.3
DMMs (*n* = 477)	15.7 ± 5.9
DHMs (*n* = 489)	24.7 ± 5.8
Pain intensity (*n* = 460)	8.9 ± 1.01
Number of failures to preventive treatments	4.2 ± 1.9
Previous treatment with topiramate	91.4%
Previous treatment with beta-blockers	79.3%
Previous treatment with amitriptyline	70.4%
Number of cycles	25.8 ± 5.7
20–32 cycles	91.5%
33–40 cycles	6.5%
>40 cycles	2%

DHMs, days of headache per month; DMMs, days of migraine per month; MOH, medication overuse headache; SD, standard deviation.

In terms of efficacy, the responder rate was 59.1% and the mean reduction in MMDs was 9.34 days (from 15.7 to 6.36 days; *p* < 0.001). The MHDs were also reduced by 14.9 days (from 24.7 to 9.8 days; *p* < 0.001). The main pain intensity (*n* = 458) was 6.3 ± 1.89. 10.4% of the patients reported acute medication overuse (*p* = 0.000). At the last visit, 47.8% of the patients underwent oral concomitant preventive treatment, most commonly amitriptyline (20%), topiramate (11.4%) and beta-blockers (10.8%) (Table 2).

**Table 2:** Efficacy parameters before and after 5 years of treatment.

Variable	Baseline	Last visit	*p*-value
	Mean (SD)/median [IQR]	Mean (SD)/median [IQR]	
DHMs (*n* = 477)	24.7 ± 5.8/27 [20–30]	9.8 ± 6.8/8 [5–12]	<0.001
DMMs (*n* = 489)	15.7 ± 5.9/15 [10–18.5]	6.3 ± 4.6/5 [3–9]	<0.001
Non-responder (%)		6.3	
Partial responder (%)		20	
Responder (%)		37.7	
High responder (%)		36	
Pain intensity (*n* = 460)	8.9 ± 1.0/9 [8–10]	6.3 ± 1.8/7 [5–8]	0.000
Non-responder (%)		11.9	
Partial responder (%)		28.8	
Responder (%)		35.4	
High responder (%)		23.9	
MOH (%)	66.4	10.4	0.000

DHMs, days of headache per month; DMMs, days of migraine per month; IQR, interquartile range; MOH, medication overuse headache; SD, standard deviation.

With regard to the side effects, 17.5% of the patients reported neck pain, 17.3% headache, 8.5% brow/eyelid ptosis, 7.5% temporal muscle atrophy and 3.2% trapezius muscle atrophy (Table 3). There were no serious AE or treatment discontinuation because of safety or tolerability issues.

**Table 3:** AE collected during the BoNTA treatment (for 5 or more years).

AE	Absolute number and percentage
Neck pain	85 (17.5%)
Headache	84 (17.3%)
Brow/eyelid ptosis	41 (8.5%)
Temporal atrophy	36 (7.5%)
Trapezius atrophy	15 (3.2%)

## Discussion

This study is the first multicentre, retrospective real-world study on patients undergoing long-term treatment of CM with BoNTA for over 5 or more years. Our results revealed an excellent tolerability profile and sustained effectiveness with a response rate of nearly 60% in a relevant number of individuals with CM who had received an average of 27 cycles with BoNTA. While the average dosages (182 U) felt in the range of the recommended after the PREEMPT clinical trials, our results show that in clinical practice they can be scheduled every 4 months in almost 3 out of 10 patients and every 6 months in a minority after several years of use.

For BoNTA, it has been suggested to stop treatment in patients who convert from CM to episodic migraine or in patients who experience fewer than 10 MHDs for at least 3 months (23). In our series, based on the excellent tolerability, we prioritize patients’ preferences.

Our findings are consistent with those of previous studies that analysed the experience with BoNTA treatment for CM over a period of up to 4 years (Table 4). In the Chronic Migraine OnabotulinuMtoxinA Prolonged Efficacy Open Label (COMPEL) study that enrolled approximately 500 adult patients with CM from international sites (10), the patients were evaluated for over 108 weeks, following a 4-week baseline period. 18.3% of patients have reported ≥1 treatment-emergent AE, most commonly neck pain (4.1%). One patient suffered a serious treatment-related AE (rash). No deaths were reported. In the REPOSE Study, which was a European, open-label, multicentre, prospective, noninterventional study (11), the patients were observed for 24 months after initiating BoNTA treatment. Overall, 18.3% of the patients suffered an adverse drug reaction; most were mild to moderate intensity, with only 1.3% of patients reporting a serious AE. Moreover, eyelid ptosis (5.4%), neck pain (2.8%) and musculoskeletal stiffness (2.7%) were the most frequently reported.

**Table 4:** Characteristics of the real-life studies of BoNTA administration for the treatment of chronic migraine.

Authors and journal	Year and country	Type of study	Patients	Follow-up period	AE
Cernuda–Morollón E, et al. (12) *Cephalalgia*	2015 Spain	Retrospective	*n* = 132	>12 months (*n* = 108) >24 months (*n* = 54) >36 months (*n* = 50) >48 months (*n* = 20)	TRAE in the first year Eyelid ptosis (5.3%) Neck pain (4.5%) Dysphagia (0.8%) Local muscle atrophy in the frontotemporal region (25%)^a^
Negro A, et al. (19) *SpringerPlus*	2015 Italy	Prospective	*n* = 155	24 months	Injection site pain (3.3%) Neck pain (3.8%) Musculoskeletal weakness (3.8%) Headache (3.7%) Eyelid ptosis (2.9%)
Blumenfeld AM, et al. (10) *Cephalalgia*	2016 United States Australia Korea	Prospective	*n* = 716	108 weeks	TRAE with an incidence of >1% Neck pain (4.1%) Eyelid ptosis (2.5%) Musculoskeletal stiffness (2.4%) Injection site pain (2%) Headache (1.7%) Muscular weakness (1.4%) Facial paresis (1.3%)
Domínguez C, et al. (15) *European Journal of Neurology*	2017 Spain	Prospective	*n* = 725	12 months	12.3% of patients presented with AEs after the first injection (10.2% were mild)
Guerzoni S, et al. (14) *Frontiers of Neurology*	2017 Italy	Retrospective	*n* = 90	36 months	Erythema (7.7%) Injection site oedema (3.3%) Itching (3.3%) Muscle weakness (3.3%) Headache (2.2%) Transitory palpebral ptosis (1.1%)
Santoro A, et al. (16) *Neurological Sciences*	2017 Italy	Retrospective	*n* = 207	18 months	Neck pain (0.9%)
Vikelis M, et al. (18) *Journal of Headache and Pain*	2018 Greece	Prospective	*n* = 65	36 months	Wheals in the injection site Shoulder and/or neck pain
Ahmed F, et al. (11) *Journal of Headache and Pain*	2019 Germany Italy Norway Russia Sweden Spain United Kingdom	Prospective	*n* = 641	24 months	Eyelid ptosis (5.4%) Neck pain (2.8%) Musculoskeletal stiffness (2.7%)
Santoro A, et al. (17) *Neurological Sciences*	2020 Italy	Retrospective	*n* = 109	48 months	Neck pain (5.8%)
Ornello R, et al. (20) *Journal of Headache and Pain*	2020 Italy	Retrospective	*n* = 115	15 months	Local tension (12.2%) Local pain (10.4%) Muscle weakness or atrophy (9.6%)

*n*, number; TRAE, treatment-related AE.

a Two of eight patients treated for more than 5 years.

In the study series of Cernuda–Morollón et al. (12), AE during the first year were reported by 14.4% of the patients, including eyelid ptosis (5.3%), cervical pain (4.5%), dysphagia (0.8%) and a mixture of these (3.8%). Moreover, one-quarter of the patients (*n* = 2) who were treated for more than 5 years showed local muscle atrophy in the frontotemporal region. Interestingly, these studies also found that in around 40% of CM patients, BoNTA injections could be delayed to 4 months after the first year.

The first one-year prospective multicentre study conducted by Domínguez et al. (15), which was performed in 13 hospitals in Spain, confirmed the effectiveness of BoNTA treatment. Only 12.3% of patients experienced AE after the first injection of BoNTA, of which 10.2% were mild. After 12 months, 94.9% of the patients reported no AEs.

In a 15-month-prospective study, at the end of follow-up, 40 of the 115 included patients (34.8%) reported mild AE, the most common being local tension (*n* = 14; 12.2%), local pain (*n* = 12; 10.4%) and muscle weakness or atrophy (*n* = 11; 9.6%) (20).

In an 18-month (six quarterly cycles) retrospective study conducted by Santoro et al. (16), the efficacy of BoNTA was evaluated at each time point, in addition to the comparison with the baseline values. They proved that repeated injections over time could result in favourable outcomes in support of the observations by Negro et al. (19). During the BoNTA exposure, the reported treatment-related AE was neck pain (4.25%). In another four-year retrospective study, the same author (17) reaffirmed the sustained benefits and good tolerability of this treatment, and only a few patients (5.8%) reported neck pain during the first year of therapy.

It should be noted that the 3-year retrospective study of Guerzoni et al. (14) analysed a total of 90 patients with CM. The most frequent AEs were transitory and localised in the injection sites, mainly due to the injection procedure rather than the BoNTA effects, specifically erythema (7.7%), injection site oedema (3.3%) and itching (3.3%). The BoNTA-dependent AEs were muscle weakness (3.3%), headache (2.2%) and transitory palpebral ptosis (1.1%). Similarly to previous studies, Vikelis et al. (18) reported the sustained therapeutic effect of BoNTA in patients with CM after 3 years of treatment (months 37–39 post-treatment initiation). In their prospective study, few cases experienced transient and mild AEs, such as wheals at the injection site and shoulder and/or neck pain.

Even though the number of patients who discontinued treatment due to poor tolerability is unknown, one of the most significant findings of this study is the confirmation of the long-term safety of BoNTA, with consistent safety and tolerability profiles observed over a period exceeding 5 years. Throughout the study, the incidence rates of adverse events remained stable, and no new adverse events emerged, indicating sustained safety.

In addition to the inherent limitations of its retrospective nature and its focus solely on patients who continued long-term BoNTA treatment after the initial response, the present study has several other limitations. We did not have the total number of patients who started BoNTA treatment and, therefore, information about the proportion of patients who discontinued treatment because of lack of efficacy and/or tolerability was not available. This means that we cannot assess the real data about efficacy and tolerability and that the generalizability of our results may be limited to patients who are responders to BoNTA treatment. Moreover, the frequency of symptomatic treatment use in the last visit was not recorded, and only limited data on quality of life when BoNTA treatment was started was obtained.

## Conclusion

Our study confirms that BoNTA is an effective, safe, and well-tolerated long-term treatment. In patients who initially responded to BoNTA treatment, efficacy and safety are maintained for more than 5 years, with no evidence of new adverse effects.

## Data availability statement

The original contributions presented in the study are included in the article/supplementary material, further inquiries can be directed to the corresponding author.

## Ethics statement

The studies involving humans were approved by Ethics Review Board of Zaragoza Health Area (GEC-ONA-2019-01) based in the Aragón Institute for Health Research (IIS Aragón), Zaragoza, Spain. The studies were conducted in accordance with the local legislation and institutional requirements. The participants provided their written informed consent to participate in this study.

## Author contributions

MN-P: Conceptualization, Data curation, Investigation, Writing – original draft. VG-Q: Data curation, Investigation, Writing – original draft. AM-V: Data curation, Investigation, Writing – original draft. EM: Data curation, Investigation, Writing – original draft, Visualization. AA: Data curation, Investigation, Writing – original draft, Visualization. GL: Data curation, Investigation, Writing – original draft. FM: Data curation, Investigation, Writing – original draft. MM: Data curation, Investigation, Writing – original draft. VM: Data curation, Investigation, Writing – original draft. DG-A: Data curation, Investigation, Writing – original draft. CG-O: Data curation, Investigation, Writing – original draft. AG-V: Data curation, Investigation, Writing – original draft. FV: Data curation, Investigation, Writing – original draft. IB: Data curation, Investigation, Writing – original draft. NM: Data curation, Investigation, Writing – original draft. JV: Data curation, Investigation, Writing – original draft. JC-L: Data curation, Investigation, Writing – original draft. JR-V: Data curation, Investigation, Writing – original draft. EC: Data curation, Investigation, Writing – original draft. PI: Conceptualization, Data curation, Formal analysis, Investigation, Supervision, Writing – original draft, Writing – review & editing. FI: Data curation, Investigation, Writing – original draft. AG-P: Data curation, Investigation, Writing – original draft. RB: Data curation, Investigation, Writing – original draft. PP-R: Data curation, Formal analysis, Investigation, Methodology, Supervision, Writing – original draft, Writing – review & editing. JP: Data curation, Formal analysis, Investigation, Methodology, Supervision, Writing – original draft, Writing – review & editing. SS-L: Data curation, Formal analysis, Investigation, Methodology, Supervision, Writing – original draft, Writing – review & editing.
